# Magnetic domains driving a Q-switched laser

**DOI:** 10.1038/srep38679

**Published:** 2016-12-08

**Authors:** Ryohei Morimoto, Taichi Goto, John Pritchard, Hiroyuki Takagi, Yuichi Nakamura, Pang Boey Lim, Hironaga Uchida, Mani Mina, Takunori Taira, Mitsuteru Inoue

**Affiliations:** 1 Toyohashi University of Technology, 1-1 Hibarigaoka, Tempaku, Toyohashi, Aichi 441-8580, Japan; 2 JST, PRESTO, 4-1-8 Honcho, Kawaguchi, Saitama 332-0012, Japan; 3 Iowa State University, Ames, Iowa 50011, United States; 4 Institute for Molecular Science, 38 Nishigonaka, Myodaiji, Okazaki, Aichi 444-8585, Japan

## Abstract

A 10-mm cavity length magnetooptically Q-switched Nd:GdVO_4_ laser was demonstrated using a single-crystalline ferrimagnetic rare-earth iron garnet film. To design the Q-switching system, the magnetic, optical, and magnetooptical properties of the garnet film were measured. The diode pumped solid-state laser cavity was constructed using a 190-μm-thick garnet film with 58% transmittance. The garnet film had maze-shaped magnetic domains, and the domain walls disappeared when a field of over 200 Oe was applied. Therefore, the polarization state of the transmitted light was modified by modulating the magnetization, and a Q-switched pulse output with a pulse width of 5 ns and peak power of 255 W was achieved in the 10-mm-long cavity. The physical limitation of the pulse width was discussed with the calculated results.

Rare-earth iron garnets (RIGs) are well known ferrimagnetic materials that are used in various communication applications such as optical isolators, microwave filters^1^, and bubble domain memories^2^. In general, RIGs have a garnet structure expressed as the general formula R_3_Fe_5_O_12_, where R indicates a rare-earth ion. Substituting elements in the R or iron site results in an interesting change in the magnetic and optical properties^3^,^4^,^5^, and variation in the substitution allows broad applications^6^,^7^ for RIGs. Despite the range of potential applications, there are few studies on magnetooptical (MO) Q-switches using RIGs. We have recently proposed the use of an RIG as a Q-switch because of its quick optical modulation speed, thin thickness to obtain sufficient changes of optical states, high transmissivity, and large MO effect in the near infrared region^8^.

For a Q-switch, Q-factor modulation of a laser cavity is required to generate high-power short laser pulses. In particular, diode-pumped solid-state micro lasers (DPSSMLs) attract much interest because they are compact (on the order of centimetres) and demonstrate high stability and efficiency^9^,^10^,^11^. To obtain giant pulse power and small timing jitter with the integrated DPSSML, active Q-switches based on electro-optical^12^,^13^ and acousto-optical effects^14^ are more suitable than passive Q-switches such as saturable absorbers. However, active Q-switches are difficult to miniaturize, and the obtained pulse width and peak power are limited. So far, the cavity length of active Q-switches has not been less than 10 mm.

To solve this issue, MO materials could be strong candidates for active Q-switches in DPSSMLs because of their ultrafast response time^15^,^16^ and their widely studied lamination techniques^17^,^18^. In 1995, Zhou *et al*. demonstrated an MO Q-switched laser with a paramagnetic material that showed a Q-switched laser pulse with a full width at half maximum (FWHM) of 100 ns^19^; however, there has been no significant progresses since this report. We recently showed the first MO Q-switched laser using RIG^8^, but its cavity length was not any shorter than other active Q-switches even though its thickness was less than 1 mm. Moreover, the detailed switching mechanism was not revealed, so the physical limitations could not be discussed.

In this study, we report an MO Q-switch using a short cavity that cannot be realized by other active Q-switches. This MO Q-switch is based on the motion of magnetic micro-domains (MMDs) in a ferrimagnetic garnet film. A single-crystalline RIG film sandwiched by two coils was installed in a diode end-pumped Nd:GdVO_4_ laser cavity, and Q-switching operation was demonstrated using the pulsed magnetic field.

## Results and Discussion

### Magnetooptical film

The 190-μm-thick single crystalline RIG film used as the MO Q-switch was fabricated on a 560-μm-thick Gd_3_Ga_5_O_12_ (GGG) substrate via liquid phase epitaxy. Both sides were polished to avoid light scattering. The magnetic, optical, and MO properties of the RIG were measured to design a pulse-field generator for Q-switching. A vibrating sample magnetometer (TM-VSM 261483-HGC, Tamakawa) and a spectrophotometer (UV-3150, Shimadzu) were used to study the magnetic and optical properties, respectively. Measurement of the Faraday rotation (FR) of the RIG was conducted using the rotating analyser method. For investigating the FR and obtaining MO images, a power meter (918D-IR-OD3, Newport) and a charge-coupled device (CCD) camera (QICAM Fast 1394, QImaging) were used as detectors, respectively. The entire measurement was conducted at room temperature.

**Figure 1:**
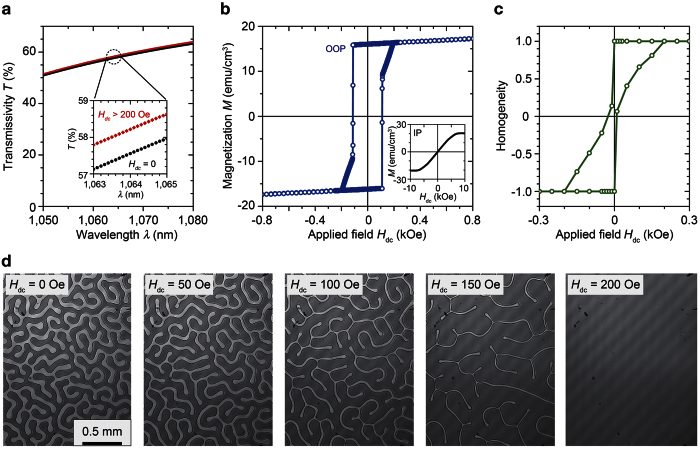
Properties of the single crystalline garnet film. (**a**) Transmittance spectrum of the magnetooptical garnet with/without the applied magnetic field. An enlarged plot in the vicinity of the wavelength of *λ* = 1,064 nm is shown in the inset. (**b**) Magnetic hysteresis loop of the garnet when the field was applied in the out-of-plane (OOP) direction. The in-plane (IP) magnetization loop is shown in the inset. (**c**) Homogeneity of the polarization plane state of the transmitted light through the garnet. (**d**) Polarization microscope images with various applied magnetic fields *H*_dc_.

Figure 1a shows the transmittance of the RIG for applied magnetic fields of 0 Oe and 200 Oe. The applied field was perpendicular to the film surface. At the wavelength of 1,064 nm, which is the fluorescent wavelength of Nd:GdVO_4_, the transmittances of the RIG measured by the spectrophotometer with applied magnetic fields of 0 Oe and 200 Oe were 57.6% and 58.2%, respectively. The refractive index *n*_MO_ and extinction coefficient *κ*_MO_ obtained by fitting software (SCOUT) based on the Fresnel equation were 2.23 and 1.03 × 10^–4^ (~ −53 dB/cm), respectively. Figure 1b shows the measured magnetic hysteresis loop. The easy axis was out-of-plane due to the crystalline anisotropy of film. The magnetization was saturated out-of-plane by an external magnetic field of over ~200 Oe, and the FR at the wavelength of 1,064 nm was ~47°. Figure 1c shows the homogeneity of the polarization state of the transmitted light obtained with the polarization microscope images shown in Fig. 1d. Maze-like magnetic domains with an average width of ~50 μm were observed in this garnet film without external magnetic field. These maze-like magnetic domains generated diffraction, showing a transmission difference of 0.6%.

### Laser set up

**Figure 2:**
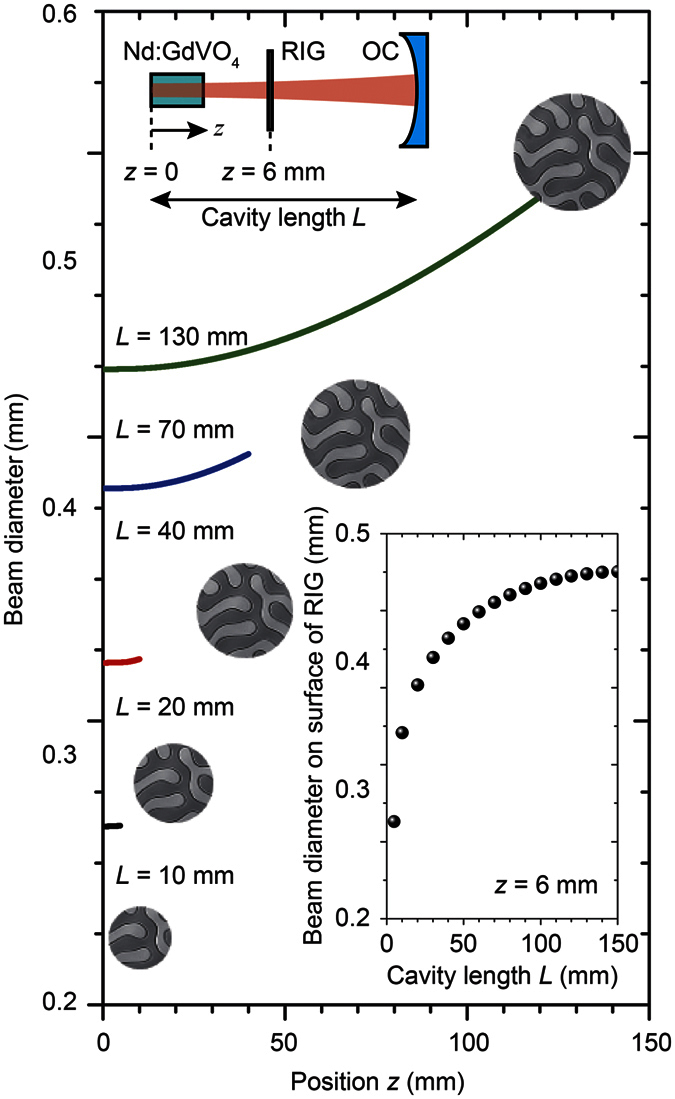
Beam diameter in the cavity with respect to the cavity length. Lasing material Nd:GdVO_4_, RIG, and OC were placed in alignment as shown in the schematic on the upper left. The RIG was placed 6 mm from the Nd:GdVO_4_ surface (*z* = 6). The beam spot sizes at the surface of RIG with various cavity lengths are expressed by the circular images of MMDs, and the diameter is plotted in the bottom right inset.

The constructed laser cavity is shown in the inset of Fig. 2. The aspects of the gadolinium vanadate crystal were 3 × 3 × 4 mm^3^ (*a*-cut and Nd 0.5 at.% doped), and the side faces were contacted with a copper heat sink, which was controlled with a Peltier module to keep the lasing material at 20 °C. The heat sink and the 300-mm curvature radius concave mirror (output coupler, OC) with a reflectance *R* of 90% at the wavelength of 1,064 nm were placed with the distance of *L*. The entire set up was fixed with a metal jig (Thorlabs). The OC can move along the rods to change the cavity length from 10 to 130 mm. This length was fixed in the Q-factor control experiment using a dc magnetic field. Pumping light at a wavelength of 808 nm was provided by a fibre-coupled continuous wave (CW) laser diode (LIMO32-F400-DL808, LIMO) and was focused on the centre of the Nd:GdVO_4_ crystal using a lens set. The lasing material was coated with dielectric mirrors: high reflectance (HR) of 99.8% at the wavelength of 1,064 nm and high transmittance (HT) of 98% at the wavelength of 808 nm on the input side. On the opposite surface of the Nd:GdVO_4_ crystal, the coating had HT of 98% at the wavelength of 1,064 nm and HR of 99.8% at the wavelength of 808 nm. The RIG was placed at 6 mm from the input surface of NdGdVO_4_. Figure 2 shows the beam diameter in the cavity estimated using a ray-trace program (Paraxia-Plus, SCIOPT) based on the physical constants of the used materials (see Methods). The spot diameter at the surface of the lasing material was 0.45 mm. On the garnet film, the spot diameter was changed as shown in the right bottom inset of Fig. 2; thus, the light transmitted through at least three magnetic domains when there was no applied field.

**Figure 3:**
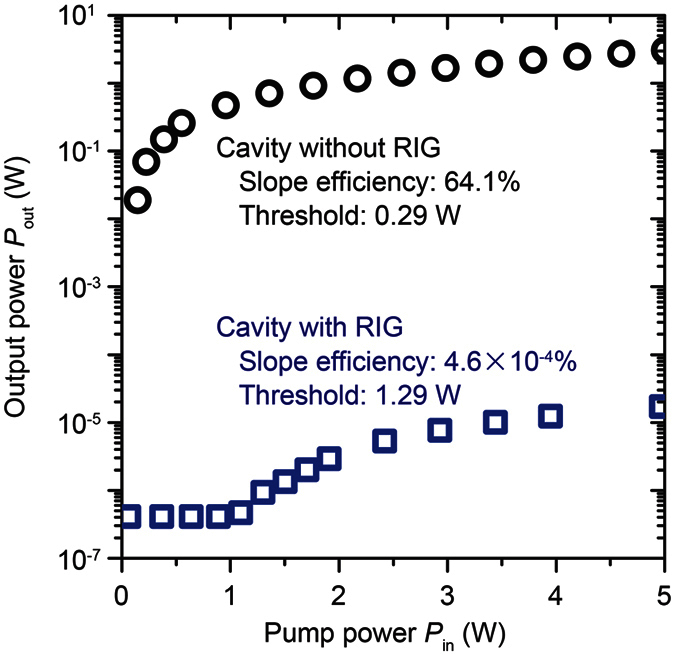
Output properties of the laser cavity with/without MO film. Output power of the laser under CW operation with respect to the pump power. Circles and squares show the output characteristics of the cavity with and without the RIG, respectively.

Figure 3 shows the measured laser output versus pump power of the used laser cavity with and without the RIG. No magnetic field was applied. The reflectance *R* of 90% showed the slope efficiency *η*_s_ of 64.1%, and the threshold power was 0.29 W. The prepared RIG was inserted into this cavity; as a result, *η*_s_ was degraded to 4.6 × 10^–4^%, and the threshold power was 1.29 W. The increased loss and degraded slope efficiency were due to the absorption of the garnet film and its surface reflection. These properties can be decreased by improving the composition and optimization of the anti-reflection coating.

### Magnetic-domain-controlled Q-factor

To understand the effect of domain motion, the Q-factor was changed by the dc field by changing the position of the ring-shaped samarium cobalt permanent magnet. The internal diameter of the magnet was 7 mm, the outer diameter was 12 mm, and the thickness was 3.5 mm. The generated magnetic field was uniform on the garnet in the beam exposure area. A sufficient magnetic field for saturating the used magnetic garnet was obtained by moving the permanent magnet. The laser properties and those for the laser containing magnetic garnet were measured.

The output power was changed as a function of the dc magnetic field via the MMD motion of the garnet film. The power was increased by two orders of magnitude by applying a saturation magnetic field of ~200 Oe. Moreover, the Q-factor measured by the spectral analyser of the output was increased from 887 to 986. This increase is due to the banishment of the magnetic domains. When the FR is 0°, the polarization plane of the light, which passed through the different domains, would be rotated to a different direction. This rotation direction depends on the magnetization direction of the passed magnetic domains. These lights are spatially overlapped and mixed in the cavity. The lack of uniformity of the polarization states of the transmitted light could cause a decrease in the Q-value of the cavity. In contrast, when the FR is over 47°, the polarization state of the transmitted light is spatially uniform; thus, the output power was drastically increased, indicating controllability of the laser power by the garnet film.

### Magnetooptical Q-switching

**Figure 4:**
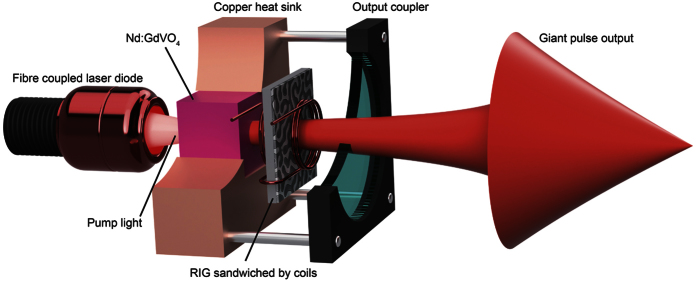
Schematic of the laser-diode end-pumped Nd:GdVO_4_ laser cavity containing the RIG sandwiched by a pair of 5.3-mm diameter coils. The lasing material was fixed in the copper heat sink. The heat sink and the OC formed a cage-shaped system with rods, and the cavity length can be changed within 10–130 mm.

Subsequently, MO Q-switching with a pulsed field was conducted in the cavity. The RIG sandwiched by coils enabled the generation of a sufficient magnetic field to saturate the magnetization of the garnet film. The diameter of the coil (~5.3 mm) was sufficiently large to allow the beam to pass through the coil. The number of turns in the coil (3 turns) was minimized to decrease the inductance that would shorten the width of the electrical pulse. This MO Q-switch set was inserted in the cavity. Figure 4 shows a schematic of the used laser cavity with the MO Q-switch setup sandwiched by coils; part of the heat sink and the OC are cut away for better visualization of the setup. The distance between the lasing material and the OC was changed, and the peak power and pulse width of the MO Q-switch were measured.

**Figure 5:**
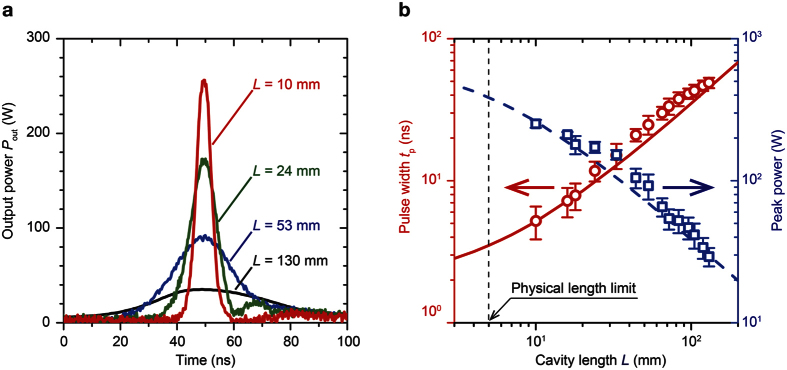
MO Q-switched pulse output characteristics. (**a**) Output pulse shapes when the cavity length was changed within 10–130 mm. (**b**) Pulse width and peak power as a function of the cavity length. The pulse width (red circles) was decreased, and the peak power (blue squares) was increased as the cavity was shortened. The error bars show the standard deviation of ten duplicate measurements. The calculated value of the pulse width (solid curve line) and peak power (dashed curve line) with the changing cavity length is also plotted. The vertical dashed line indicates the physical length limit of the cavity because of the total thickness of the used Nd:GdVO_4_ and RIG (*L* = 4.75 mm).

The pulsed field for Q-switching was generated by a pulsed current with an amplitude of 56 A and width of 2.5 μs. The obtained Q-switched pulse from the 130-mm-long cavity had a peak power of 30 W and FWHM of 45 ns. This was comparable with our previous report^8^. The cavity length was changed from 130 mm to 10 mm. When the cavity is shortened, reduction of the pulse width and an increase of the peak power was achieved, as shown in Fig. 5a. Figure 5b shows the change of the output pulse width and peak power when the cavity length was varied. The pulse width proportionally decreased with the cavity length, and the peak power was inversely proportional to the decreasing cavity length. The shortest pulse width *t*_p_ was 5 ns, and the largest peak power was 255 W; thus, 1.3 μJ/pulse. The repetition rate was 100 Hz. The peak power of 255 W was 3.4 × 10^4^ times larger than that under CW operation.

### Discussion with theoretical analysis

**Figure 6:**
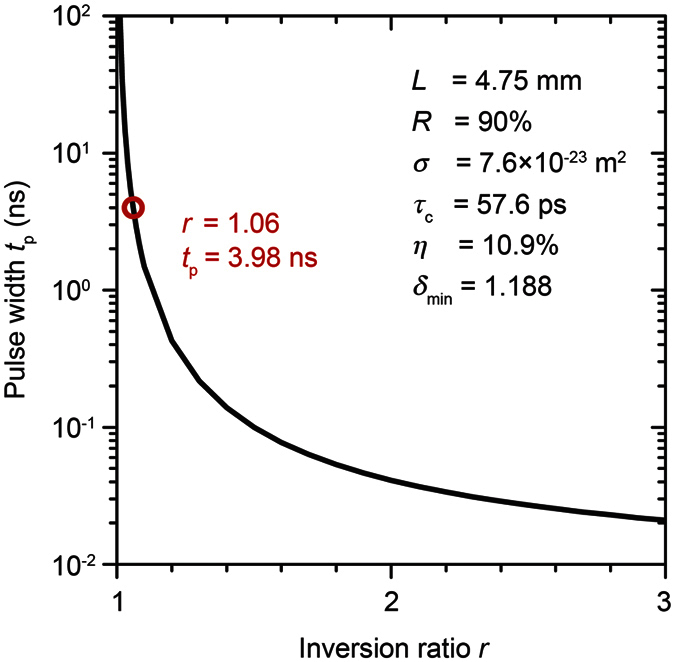
Pulse width *t*_p_ estimation based on the rate equation of the Q-switched laser including RIG. The red circle indicates the value of the inversion ratio and pulse width at the physical cavity length limit (*L* = 4.75 mm). The curved line plots the calculated dependence on various inversion ratios *r* using the cavity parameters shown in the inset. An increase of *r* can improve the pulse width.

The measured pulse width *t*_p_ can be written as^20^,^21^


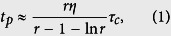


where *r* is the inversion ratio, *η* is the energy extraction efficiency, and *τ*_c_ is the round-trip time in the cavity. Here, *r* and *η* can be described as *r* = *N*_i_/*N*_th_ and *η* = (*N*_i _− *N*_f_)/N_i_, respectively, where *N*_i_ is the initial population inversion, *N*_th_ is the threshold population inversion, and *N*_f_ is the final population inversion. Moreover, *N*_i_, *N*_th_, *N*_f_, and *τ*_c_ can be described by *N*_i_ = *δ*_max_/(2*σL*), *N*_th_ = *δ*_min_/(2*σL*), *N*_f_ = *N*_i_ − *N*_th_·ln(*N*_i_/*N*_f_), and *τ*_c_ = 2 *L*/(*cδ*_min_), respectively, where *δ*_max_ (Q-switch is “OFF”) and *δ*_min_ (Q-switch is “ON”) are, respectively, the maximum and minimum cavity losses, *σ* is the stimulated-emission cross-section of Nd:GdVO_4_ ( = 7.6 × 10^−23 ^m^2^), and *c* is the velocity of light ( = 3.0 × 10^8^ m/s). The minimum cavity loss *δ*_min_ can be derived with the equation *δ*_min_ = −ln(*R*) − 2ln(*T*_on_), where *R* is reflectance of OC ( = 0.90) and *T*_on_ is the transmittance of RIG when a magnetic field of 200 Oe was applied (*T*_on_ = 0.582); thus, *δ*_min_ = 1.188. The maximum cavity loss *δ*_max_ was derived analytically with the measured *t*_p_ shown in Fig. 5b. The average *δ*_max_ within *L* = 10–130 mm was 1.258. These derived physical constants can be used to estimate the pulse width *t*_p_ as a function of the inversion ratio *r*. In Fig. 6, the circle shows the shortest pulse width estimated when the cavity length is at its physical length limit (*L* = 4.75 mm). As the solid line shows, to improve this MO Q-switched laser, the inversion ratio *r* needs to be increased. Hence, an increase of *δ*_max_ should contribute to the improvement of the MO Q-switched laser.

## Conclusion

We have reported that MMDs in a single-crystalline rare-earth-substituted iron garnet film caused Q-factor modulation in a laser cavity. The demonstrated active MO Q-switched laser with 10-mm cavity length provided a maximum peak power of 255 W with FWHM of 5 ns at a repetition rate of 100 Hz using a 190-μm-thick RIG film. This is the largest power and shortest pulse width obtained by MO Q-switches. In addition, the thickness of the Q-switch is extremely small in comparison with other reported active and passive Q-switches, which allowed us to construct the shortest active Q-switched laser system to our knowledge. Because the RIG could be epitaxially grown on lasing materials, we find the Q-switch system to be very promising for achieving actively Q-switched DPSSMLs, which have an even shorter cavity and a higher power output. Such MMDs can be controlled actively^22^ and quickly^23^, hence MO Q-switched laser can show novel functionalities in the near future.

## Methods

### Optical constants used in beam diameter characterizations

The used physical constants for the beam diameter calculation discussed with Fig. 2 are shown. The refractive indices of Nd:GdVO_4_, the GGG substrate, and the RIG film were 2.19, 1.94, and 2.23, respectively, and the thicknesses were 4 mm, 0.56 mm, and 0.19 mm, respectively. The curvature radius of the concave mirror (OC) was 300 mm.

## Additional Information

**How to cite this article**: Morimoto, R. *et al*. Magnetic domains driving a Q-switched laser. *Sci. Rep.* **6**, 38679; doi: 10.1038/srep38679 (2016).

**Publisher's note:** Springer Nature remains neutral with regard to jurisdictional claims in published maps and institutional affiliations.
